# Postmortem findings in Magellanic penguins (*Spheniscus magellanicus*) caught in a drift gillnet

**DOI:** 10.1186/s12917-020-02363-x

**Published:** 2020-05-24

**Authors:** Ana Carolina Ewbank, Carlos Sacristán, Samira Costa-Silva, Marzia Antonelli, Janaina R. Lorenço, Guilherme A. Nogueira, Mariana B. Ebert, Cristiane K. M. Kolesnikovas, José Luiz Catão-Dias

**Affiliations:** 1 Laboratory of Wildlife Comparative Pathology, Department of Pathology, School of Veterinary Medicine and Animal Sciences, University of São Paulo, São Paulo, SP 05508-270 Brazil; 2 Associação R3 Animal, Florianópolis, SC 88061-500 Brazil; 3 Laboratory of Wildlife Parasitology (LAPAS), Parasitology Department, Biosciences Institute, São Paulo State University (UNESP), Botucatu, SP 18618-000 Brazil

**Keywords:** Brazil, Bycatch, Drowning, Fisheries, Histopathology, Seabirds, Stranding

## Abstract

**Background:**

Penguin interaction with gillnets has been extensively reported in the Atlantic and Pacific Oceans, and is considered a major conservation threat. Among penguin species, Magellanic penguins (*Spheniscus magellanicus*) are currently considered of great concern, particularly in Brazil, where they are highly susceptible to gillnet bycatch. Nevertheless, information about drowning-associated microscopic findings in penguins is limited.

**Results:**

We describe the anatomopathological findings of 20 Magellanic penguins that drowned after getting entangled in a drift gillnet while wintering along the Brazilian shelf and washed ashore still enmeshed in Santa Catarina, Brazil. All 20 birds (19 juveniles and 1 adult; 18 females and 2 males) were in good body condition. Major gross findings were abrasion, bruising, and local erythema and edema of the wings, multiorgan congestion, jugular vein engorgement, pulmonary edema and hemorrhage, splenomegaly and hepatomegaly, fluid in the trachea, serous bloody fluid in the lungs, gastrointestinal parasites (nematodes, cestodes and trematodes), and debris in the stomach. The most common histopathological findings were cerebral and pulmonary congestion, pulmonary edema, splenic histiocytosis, lymphoid splenic hyperplasia, acute splenitis, extramedullary hepatic hematopoiesis, and parasitic enteritis. Although unspecific, the observed multiorgan congestion and pulmonary edema are consistent with previous reports of drowning in birds and may be indicative of this process.

**Conclusions:**

Drowning may be a challenging diagnosis (e.g., carcass decomposition, predation), but must be considered as a differential in all beach-cast seabird postmortem examinations. To the authors’ knowledge this is the largest anatomopathological study based on microscopic examination in drowned penguins.

## Background

Bycatch refers to incidental mortality and/or injury in fishing gear of individuals of target species that are discarded (“discarded catch”) and of nontarget species (“incidental catch”) [1–3]. Seabird bycatch mortality, in both commercial and artisanal fishing operations, is recognized as a major threat to the conservation of seabird species worldwide [4–6]. Penguin bycatch has been largely reported in the Atlantic and Pacific Oceans, in 14 of the 18 penguin species [7], and is a threat to penguin conservation. In South America, gillnet bycatch of Magellanic penguins (*Spheniscus magellanicus*) has been reported in Brazil [8–11], Argentina [12], Chile [13, 14] and Peru [15], while bycatch by trawl fisheries has been reported in Argentina [16–19]. In addition, Magellanic penguins presenting postmortem signs of fishing interaction or entanglement in fishing gear have been reported in Chile [20] and Brazil [21, 22].

Magellanic penguins are widely distributed along the southern coast of South America [23]. With an estimated global population between 1.1 and 1.6 million pairs, this species is currently listed as ‘near threatened’ due to marine pollution, fisheries bycatch and competition for food resources, habitat degradation, diseases, and climate changes [23, 24]. On the Atlantic coast, the species breeds from Northern Patagonia (Complejo Islote lobos, 41°26′S, 65°01′W) to Tierra del Fuego (54°54′S, 67°23′W), Argentina [25, 26]. During the non-breeding period (austral autumn and winter), animals from colonies at the Atlantic/Argentinean coast migrate through the continental shelf off the coast of northern Argentina, Uruguay, and southern Brazil following the Argentine anchovy (*Engraulis anchoita*), their diet’s main food item in the wintering grounds [10, 27]. Bycatch has been suggested as a significant source of mortality for this species during migration [9]. Magellanic penguins are considered highly susceptible to gillnet bycatch in Brazil, Uruguay, Argentina and Chile [7].

Drowning is defined as death following respiratory impairment consequent of submersion or immersion (partial submersion) in liquid [28, 29]. There are no pathognomonic postmortem lesions for the diagnosis of drowning in humans and in other mammals [30]. The diagnosis of death by drowning in seabirds is particularly challenging, not only due to the difficulty in differentiating drowning and postmortem submersion [31], but also because drowning studies in seabirds are scarce, and have been primarily based on beach-cast carcasses presenting unspecific external lesions compatible with alleged entanglement in fishing nets (e.g., abrasions, subcutaneous bruising) [14, 20], on the presence of water in the lungs and/or air sacs [32–35], or solely based on visual observation of the drowned bird entangled in fishing gear [36]. Furthermore, these studies are usually performed in beach-cast carcasses that have been subjected to a variety of postmortem challenges (e.g., predation, weather exposure, amount of time the carcass remained adrift, and autolysis).

Herein we describe and discuss the gross and microscopic findings of 20 Magellanic penguins that drowned after getting entangled in a drift gillnet in southern Brazil, and discuss the potential ecological effects of bycatch on Magellanic penguin conservation. Additionally, we briefly suggest data collection measures that would greatly contribute to the study of bycatch and drowning-associated lesions in seabirds.

## Results

### Macroscopic findings

**Fig. 1:**
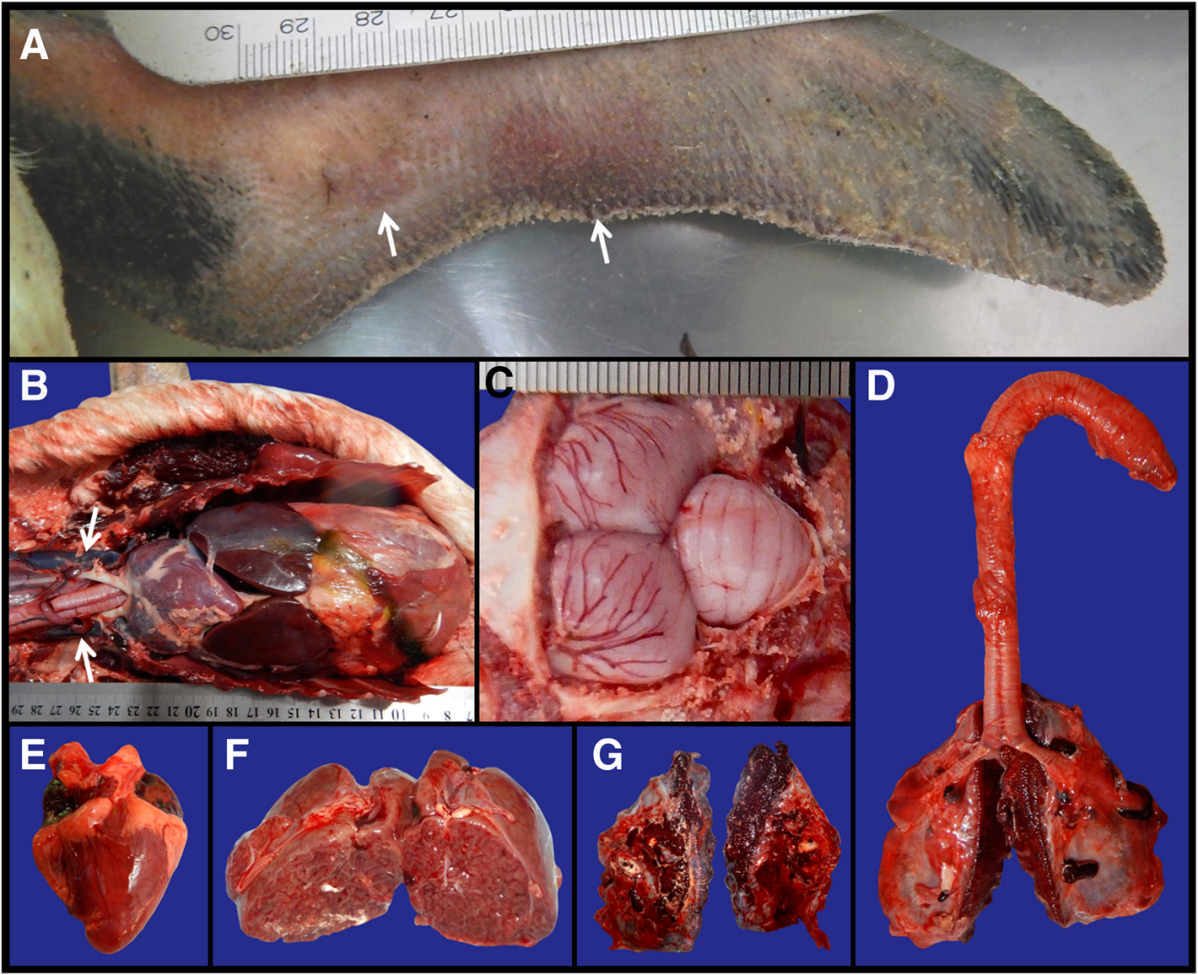
Macroscopic findings on the studied Magellanic penguins (*Spheniscus magellanicus*). All animals presented congestion in at least one system, likely caused by decreased cardiac output due to increased vascular resistance in the lungs, cardiac anoxia and acidosis. **a** Left wing (ventral view): edema and bruising (white arrows). **b** Coelomic cavity: generalized congestion and hepatomegaly, and bilateral jugular vein engorgement (white arrows). **c** Brain: congestion and edema. **d** Trachea, bronchi and lungs: congestion, and pulmonary and serosal edema. **e** Heart: normal fat deposits, congestion (especially in the left and right atria). **f** Cranial kidney lobe (transversal section): congestion. **g** Lungs: pulmonary congestion and edema

All 20 birds - 19 juveniles (18 females and 1 male) and 1 adult (female) were in good body condition. Major macroscopic findings were abrasion, bruising, and local erythema and edema of the wings (10/20; 50%), congested lungs (20/20; 100%), brain (20/20; 100%), spleen (7/20; 35%), heart (6/20; 30%), kidneys (4/20; 20%), liver (1/20; 5%), and pulmonary edema (20/20; 100%) (presence of foamy serosanguineous fluid when the lungs were cut, severely and diffusely increased lung volume and presence of a gelatinous and sanguineous layer of approximately 2 mm covering the dorsal lung surface), splenomegaly (11/20; 55%) and hepatomegaly (4/20; 20%) (Fig. 1). Jugular vein engorgement (bilateral) was not systematically recorded but was observed in at least ten animals (10/20; 50%) (Fig. 1).

Gastrointestinal parasitosis was observed in 18 out 20 penguins: nematodes *Contracaecum pelagicum* in the stomach (*P* = 80%; MII = 4.6 ± 2.2; range = 2–10 parasites per host]*,* and cestodes *Tetrabothrius* [*Neotetrabothrius*] *lutzii* [*P* = 55%; MII = 1; range = 1 parasite per host) and trematodes *Cardiocephaloides physalis* [*P* = 35%; MII = 1.8 ± 1.1; range = 1–4 parasites per host] in the intestines. Two birds presented debris in the stomach (2/20; 10%): a small nylon fragment and an unidentified piece of plastic. Gross findings are listed in Table 1.

**Table 1:** Number of Magellanic penguins (*Spheniscus magellanicus*) presenting gross pathological and histological findings listed by system and prevalence (P)

System	Macroscopic findings (***n*** = 20)	P	Microscopic findings (***n*** = 20)	P
**Cardiovascular System**	Cardiac congestion	30% (6)	Cardiac congestion	15% (3)
	Cardiomegaly	5% (1)		
	Dilated atria	5% (1)		
	Jugular vein engorgement (bilateral)	50% (10)		
**Respiratory System**	Pulmonary edema	100% (20)	Pneumonia	55% (11)
	Pulmonary congestion	100% (20)	- Mild to moderate acute diffuse granulocytic pneumonia	35% (7)
	Pulmonary hemorrhage	20% (4)	- Acute diffuse pneumonia	20% (4)
	Tracheal hemorrhage	5% (1)	Mild to moderate pulmonary congestion	40% (8)
	Tracheal blood cloth	5% (1)	Mild to moderate pulmonary edema	35% (7)
	Frothy fluid in the trachea	5% (1)	Mild tracheal congestion	5% (1)
			Mild to moderate tracheal edema	5% (1)
			Mild segmented mononuclear tracheitis	5% (1)
			Distension of the tracheal lymphoid tissue	5% (1)
			Mild extension of the tracheal lamina propria	5% (1)
**Gastrointestinal Tract**	Parasitosis (Nematode, *Contracaecum pelagicum*)	75% (15)	Acute diffuse mild esophagitis	15% (3)
	Parasitosis (Trematode, *Cardiocephaloides physalis*)	55% (11)	Mild to moderate multifocal coalescent esophageal microabscesses	10% (2)
	Parasitosis (Cestode, *Tetrabothrius lutzi*)	40% (8)	Mild enteric congestion	5% (1)
	Intestinal congestion	25% (5)	Mild to moderate acute diffuse granulocytic proventricular infiltrate	5% (1)
	Stomach congestion	15% (3)	Mild to moderate acute diffuse proventriculitis	5% (1)
	Marine debris	10% (2)	Proventricular ulcer	5% (1)
			Mild to moderate acute diffuse ventricular infiltrate	5% (1)
			Parasitic enteritis	65% (13)
			Parasitic typhlitis	20% (4)
			Coccidiosis	5% (1)
**Hepatobiliary System**	Hepatomegaly	20% (4)	Hepatic extramedullary hematopoiesis	35% (7)
	Hepatic congestion	5% (1)	Mild to moderate lymphoplasmacytic hepatitis	25% (5)
			Mild mixed multifocal hepatitis	5% (1)
			Mild predominantly mononuclear periportal hepatitis with rare granulocytes	5% (1)
			Mild perivascular lymphocytic hepatitis	5% (1)
			Granulocytic hepatitis	5% (1)
			Mild to moderate diffuse hepatic congestion	15% (3)
			Hepatic duct hyperplasia	10% (2)
			Hepatic hemosiderosis	10% (2)
			Mild to moderate diffuse vacuolar hepatic degeneration	5% (1)
**Central Nervous System**	Brain congestion	100% (20)	Cerebral congestion	95% (19)
	Meningeal congestion	25% (5)		
	Skull cap congestion	10% (2)		
**Lymphoid System**	Splenomegaly	55% (11)	Mild to moderate lymphoid splenic hyperplasia	55% (11)
	Splenic congestion	35% (7)	Mild to moderate splenic histiocytosis	35% (7)
	Congestion of the bursa of Fabricius	5% (1)	Mild to moderate acute diffuse splenitis	35% (7)
			Mild splenic congestion	10% (2)
			Mild splenic lymphocytolysis	10% (2)
			Moderate splenic lymphoid proliferation	5% (1)
			Hyperplasia of the splenic germinative centre with arteriole displacement	5% (1)
			Necrosis of the splenic germinative centre	5% (1)
**Genitourinary System**	Renal congestion	20% (4)	Renal congestion	40% (8)
			Interstitial nephritis	10% (2)
			Histiocytic lymphoplasmacytic multifocal nephritis	5% (1)
**Musculoskeletal System**	Bilateral scapulohumeral joint edema	5% (1)	NA^a^	NA
**Integumentary System**	Abrasion, bruising, and local erythema and edema of the wings	50% (10/20)	NA^a^	
**Miscellaneous findings**	Generalized hemoglobin impregnation	3/20	Pancreatic congestion	10% (2)
	Caseous material in the mesentery	5% (1)	Harderian gland congestion	5% (1)
			Thyroid congestion	5% (1)
			Parathyroid congestion	5% (1)

^a^NA: not available

### Microscopic findings

**Fig. 2:**
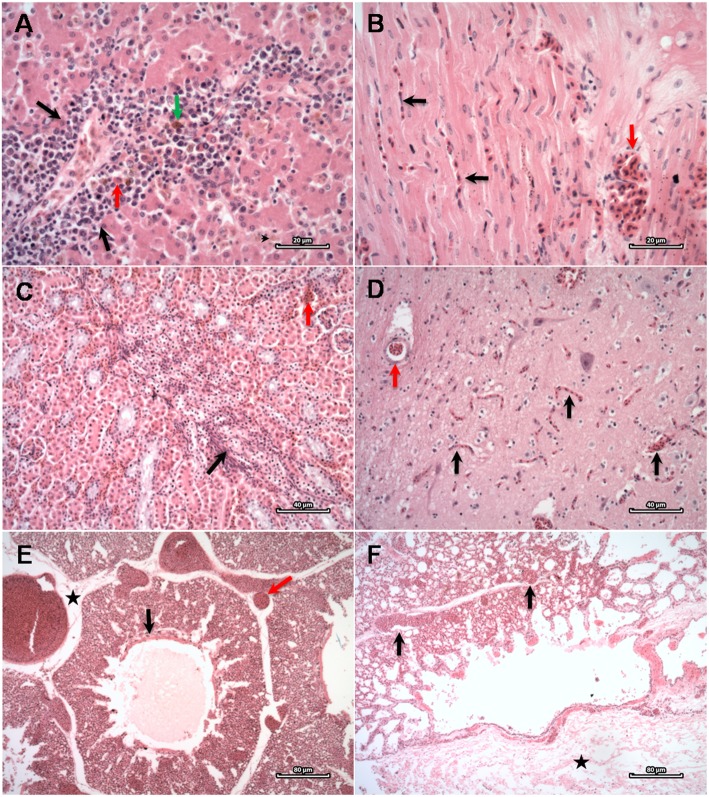
Microscopic findings on the studied Magellanic penguins (*Spheniscus magellanicus*). **a** Liver: hepatitis (red arrow), hemosiderin inside Kupffer cells (green arrow) and hepatocytes (black arrow head), extramedullary hematopoiesis (black arrows). HE. Scale bar = 20 μm (**b**) Heart: diffuse congestion (black arrows), markedly congested vessel (red arrow). HE. Scale bar = 20 μm. **c** Kidney: diffuse congestion (red arrow), multifocal interstitial nephritis (black arrow). HE. Scale bar = 40 μm. **d** Brain: diffuse congestion (black arrows) and inside arteriole (red arrow). HE. Scale bar = 40 μm. **e** Lung: congestion and edema, proteinaceous material in parabronchial lumen (black arrow), stretched interparabronchial septa due to edema (asterisk) and markedly congested vessels, including septal arterioles (red arrow). HE. Scale bar = 80 μm. **f** Lung: diffuse congestion (black arrows), markedly stretched visceral pleura (asterisk) due to edema. HE. Scale bar = 80 μm

The following mild to moderate histopathological findings were most commonly observed: cerebral and meningeal congestion (19/20; 95%), pulmonary congestion (8/20; 40%), diffuse acute granulocytic pneumonia (7/20; 35%), pulmonary edema (7/20; 35%), cardiac congestion (3/20; 15%), splenic histiocytosis (7/20; 35%), lymphoid splenic hyperplasia (11/20; 55%), acute diffuse splenitis (7/20; 35%), lymphoplasmacytic portal hepatitis (5/20; 25%), hepatic extramedullary hematopoiesis (7/20; 35%), and renal congestion (8/20; 40%). We also observed parasitic enteritis and typhlitis (13/20; 65% and 4/20; 20%, respectively). All histopathological findings are listed in Table 1 and the most significant ones are shown in Fig. 2.

Finally, our necropsy findings did not indicate the presence of metabolic or neoplastic conditions; therefore, no further diagnostic analysis were pursued.

## Discussion

**Fig. 3:**
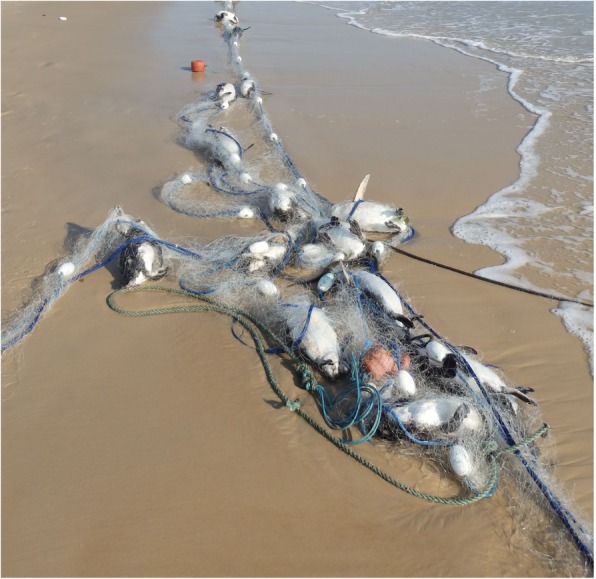
Drowned Magellanic penguins (*Spheniscus magellanicus*). Twenty birds were washed ashore still entangled in an artisanal triple-layered drift gillnet in Santa Catarina state, Brazil

This study describes the gross and histopathological findings in 20 well-preserved stranded dead Magellanic penguins washed ashore still enmeshed in an artisanal triple-layered drift gillnet (mesh size 35–45 mm). Based on our findings, we consider entanglement and subsequent drowning as the most likely explanation for the presence of these penguins in the net, once other possibilities (e.g., the animals drifted into a net when already dead) are considered remote. We hypothesize the drift gillnet was likely discarded by the fishermen because the animals were severely entangled (see Fig. 3) and, most of all, to avoid any legal responsibilities. Drift gillnets are highly efficient artisanal fishing apparatus projected for stationary use in coastal waters [37]. However, they act as a wall that ends up capturing non-target species as well (e.g., sea turtles, cetaceans, seabirds and non commercial fish species) [37–39]. As air-breathing pursuit divers, penguins are likely to interact with gillnets when foraging, transiting or resting on the surface [13–15]. Their potential impact in regards to bycatch may not be comparable with fishing nets used by commercial fisheries (e.g., longer and used offshore); however, gillnets are still a threat to penguins. Previous reports evidenced the gillnet bycatch of a large number of penguins in southern Brazil [8–11]; nonetheless, these studies did not perform complete postmortem examinations.

Aspiration of saltwater, which is 3 to 4 times more hypertonic than blood, leads to the development of acute alveolar edema as a result of protein-rich fluid drainage from the intravascular space into the pulmonary alveoli, disrupting the integrity of the alveolar–capillary membrane and increasing its permeability, exacerbating fluid, plasma, and electrolyte shifts, and causing dilution and washout of surfactant [28, 29, 40]. When fluid enters the airways, it usually results in bronchospasm, leading to an increase in relative intrapulmonary shunting [28, 41]. These physiopathological events combined with the consequent decreased lung compliance, atelectasis, and ventilation-perfusion mismatch result in hypoxemia, and ultimately death [28, 29, 41]. Meanwhile, the acute hypoxemia and stress response release high levels of catecholamine, leading to transient tachycardia and hypertension [42]. Hypoxemia may also reduce myocardial contractility and along with acidosis increase the risk for arrhythmias (e.g., ventricular tachycardia, fibrillation, and asystole) and cardiogenic shock [42]. Hypoxia intensifies as the arterial oxygen tension decreases, leading to bradycardia and vasoconstriction in ‘nonvital’ organs to promote blood flow to the heart and central nervous system and reduce myocardial oxygen consumption, in an attempt to prolong hypoxia tolerance. Additionally, during this process, endothelial cells are activated, releasing inflammatory mediators that contribute to the development of a systemic inflammatory response syndrome often accompanied by peripheral vasoplegia and capillary leakage [28].

The congestion of multiple organs (upon microscopy, more often seen in the brain, lungs and kidneys) and the pulmonary edema observed in both macroscopic and microscopic examinations of our cases ratify the findings of Vanstreels et al. [22] and Simpson and Fisher [43] in seabirds, and were strongly suggestive of drowning. Generalized congestion was likely caused by increased pulmonary vascular resistance, cardiac dysfunction secondary to the extent and duration of hypoxia, and changes in acid–base balance (acidosis), that lead to decreased cardiac output [22, 28, 42], whereas pulmonary edema was caused by aspiration of saltwater and/or increased pulmonary venous pressure [28, 29]. To the authors’ knowledge, this is the largest study based on gross and microscopic pathology in penguins. Only recently, Vanstreels et al. [22] reported the gross and microscopic findings of two Magellanic penguins that drowned and died after getting entangled in gillnet in southeastern Brazil: one in a floating (3 m) and the other in a bottom gillnet (12 m). Some of the previous studies with seabirds have relied solely on gross findings [43] or on histopathological examination of samples partially compromised by freezing [20, 32] to diagnose drowning, when microscopic examination is essential for a more accurate diagnosis.

Other significant gross and/or microscopic findings, useful to evaluate the health of migrating penguins, but likely not related with drowning, were pneumonia, splenomegaly and hepatomegaly, splenic histiocytosis and lymphoid hyperplasia, hepatic extramedullary hematopoiesis, and gastrointestinal parasitosis (associated with enteritis and typhlitis). Even though the pneumonia findings were characterized as acute, the drowning event was fatal and could not have lasted long enough to promote such condition, in spite of the lung inflammation seen in near-drowned cases with seawater inhalation [44]. Granulocytic pneumonia was previously described in two drowned penguins [22]. We consider that the etiology of the pneumonia seen in our study was not related to the drowning event and that its causes remain unknown. The observed splenomegaly and hepatomegaly could be due to a combination of one or more of the following factors: congestion, increased antibody production caused by parasite infection/infestation [45], and immunological challenges (i.e., marine pollution and infectious agents) [46–48]. The observed splenic histiocytosis and lymphoid hyperplasia could also have been caused by parasite infection/infestation, and immunological challenges - commonly faced by this species during migration. Hepatic extramedullary hematopoiesis - the formation and development of blood cells in organs other than the medullary spaces of the bone marrow [49], was also observed in the liver of five penguins. This finding may be associated with immune response following infection and exercise, or with increased erythropoietin production during hypoxia [49, 50]. We believe the immune response to parasite infection and physical challenge required by migration was possibly the causative factor, once these birds went through an acute hypoxic event (drowning) that was not long enough to increase erythropoietin production. Gastric parasitosis caused by the nematode *C. pelagicum*, and mild to severe multifocal to diffuse intestinal parasitosis caused by the cestode *T.* (*Neotetrabothrius*) *lutzi* and the trematode *C. physalis* were also observed. The described parasite species likely caused the parasitic enteritis and typhlitis reported here, and are commonly reported in Magellanic penguins; however, most of these studies are primarily taxonomic and only a few have related their presence to diseases in penguins [51–53]. Although parasitosis may strongly influence the physical condition of their hosts, and therefore, increase their vulnerability [54], all these animals were in good body condition. The impact of parasites on Magellanic penguins remains unclear, requiring further extensive pathological studies to clarify parasitic influence on this species.

All birds had empty stomachs upon necropsy, with the exception of two juveniles (2/20; 10%) that presented a small amount of marine debris in their stomachs (Table 1). The empty stomachs could be explained by the following hypothesis: (1) entanglement occurred as soon as the penguins started interacting with the gillnet, leaving them no time to ingest any prey or (2) the birds got entangled while transiting or resting at sea. Empty stomachs are often described in drowned seabirds [14]. Studies on Magellanic penguin carcasses washed ashore in southern and southeastern Brazil reported that, respectively, 14.86% (26/175) [55] and 80% (115/144) [21] of the individuals presented materials of anthropogenic origin in the stomach (marine debris). Marine debris ingestion by Magellanic penguins poses a threat for the species’ conservation throughout its distribution and has been widely reported in Brazil [55–59]. The discussed hypothesis for such findings include juvenile inexperience, hunger, and difficulty in finding food (e.g., reduced diving capacity due to poor physical condition) [55, 60]. However, both birds were in good body condition and likely ingested the debris involuntarily, either by mistake or in the body of a prey [55].

The majority of the Magellanic penguins evaluated in this study were juveniles, and similarly to the only adult, were in good body condition – suggesting that the observed histopathological processes were not compromising the birds’ ability to successfully feed and meet their daily energy requirements. Most of the juveniles found ashore in Brazil are emaciated or cachectic [57, 61]; thus, our microscopic study of apparently healthy wintering individuals provides valuable anatomopathological information regarding this poorly studied phase of their life cycle. The age groups described in previous reports of penguin bycatch in Brazil are contradictory. Vanstreels et al. [22] and the present study reported mostly juveniles, respectively, 100 and 95%, while Cardoso et al. [9], Marques et al., 2018 [10], and Fogliarini et al., 2019 [11] identified mostly adults (respectively, 81, 77 and 53%).

We found a higher number of females than males in this study, likely as a consequence of sexual dimorphism-related mechanisms that determine sexual differences in spatial domains [62, 63] and habitat segregation between Magellanic penguin sexes during wintering dispersal. Age and sex parameters are important factors in the assessment of bycatch impacts [62]. Sex ratio variation in wild populations has important consequences for population dynamics by compromising population viability [63]. The age- and sex-related data of Magellanic penguin bycatch in wintering areas is still limited; however, long-term situations affecting both juveniles and females (as seen in this study) could potentially reduce population productivity.

## Conclusions

To the authors’ knowledge, this is the largest anatomopathological study based on microscopic examination in drowned Magellanic penguins. Drowning must be considered a differential diagnosis in all beach-cast seabird postmortem examinations, especially in those presenting generalized congestion and edema. This diagnosis should be established based on gross and microscopic findings, preferably associated with recovery history (when present), and must be promptly and consistently performed by trained personnel, and fully registered through photo identification to further characterize the physiopathology and postmortem findings of drowning. Finally, in order to characterize seabird bycatch events, including in Magellanic penguins, and understand the impact of fishing interaction on their conservation, future studies need to identify the age, sex and stage of the life cycle in which the birds were at the time of the event (e.g., breeding season, migration), and interpret such findings based on the species’ conservation status, population size, demography, dispersal at sea, and estimates of bycatch mortality throughout the species’ range.

## Methods

### Samples

On August 17th 2016, 20 Magellanic penguins that drowned after being caught in an artisanal triple-layered drift gillnet (Fig. 3) during wintering were washed ashore, still entangled, at Praia dos Ingleses (27°26′ S, 48°23′ W) in Florianópolis county, Santa Catarina state, southern Brazil. The carcasses were immediately collected and necropsied by Associação R3 Animal/Coastal Monitoring Program – Santos Basin (Projeto de monitoramento de Praias da Bacia de Santos - PMP-BS). The necropsy regimen was conducted within the following time frame: five carcasses in 24 h, ten in 48 h and five in 72 h. Carcasses not necropsied on the same day were kept refrigerated until necropsy. All carcasses appeared very fresh and had no signs of predation. Necropsies were performed in fresh or refrigerated carcasses within 72 h following their discovery, according with previously established techniques [64, 65]. Upon necropsy, body condition was determined based on palpation and posterior visualization of pectoral muscles and the keel [48]. Age was determined based on plumage characteristics [66] and sexing was established upon visualization of the gonads [48]. All samples used in this study were collected in full compliance with specific federal permits issued by the Brazilian Ministry of Environment and approved by the Biodiversity Information and Authorization System (SISBIO 20825–8) and by the Brazilian Institute of Environment and Renewable Natural Resources – IBAMA (ABIO 640/2015). Because this study involved dead birds, it did not require official or institutional ethical approval.

### Histopathological study

Collected tissue samples (heart, aorta, trachea, lungs, brain, thyroid, parathyroid, tongue, esophagus, proventriculus, intestines, liver, spleen, gall bladder, pancreas, kidneys, skeletal muscle, bursa of Fabricius, Harderian gland, uropygeal gland and supraorbital gland) were placed in 10% buffered formalin. Formalin-fixed tissues were routinely processed, sectioned at 5 μm, stained with hematoxylin and eosin, and examined under light microscopy.

### Parasitological identification

Parasite samples were recovered from the gastrointestinal tract of 18 out of 20 penguins, and prepared for morphological identification according with the group (nematode, trematode or cestode) and following standard protocols [67], and identified based on measurements and morphological observations under light microscopy on a computerized system of image analysis (Qwin Lite 3.1, Leica Microsystems), according to specific literature [52, 68–70]. The prevalence (P), mean intensity of infection (MII) and standard deviation were calculated according to Bush et al. [71] in the program Bioestat 5.3.

## Data Availability

The datasets of the current study are available from the corresponding author upon request.
